# Integrative Multiomics and Regulatory Network Analyses Uncovers the Role of OAS3, TRAFD1, miR-222-3p, and miR-125b-5p in Hepatitis E Virus Infection

**DOI:** 10.3390/genes14010042

**Published:** 2022-12-23

**Authors:** Sonam Gupta, Prithvi Singh, Alvea Tasneem, Ahmad Almatroudi, Arshad Husain Rahmani, Ravins Dohare, Shama Parveen

**Affiliations:** 1 Molecular Virology Laboratory, Centre for Interdisciplinary Research in Basic Sciences, Jamia Millia Islamia, New Delhi 110025, India; 2 Mathematical and Computational Biology Laboratory, Centre for Interdisciplinary Research in Basic Sciences, Jamia Millia Islamia, New Delhi 110025, India; 3 Department of Medical Laboratories, College of Applied Medical Sciences, Qassim University, Buraydah 51452, Saudi Arabia

**Keywords:** hepatitis E virus, WGCNA, FFL, protein-protein docking, PPI

## Abstract

The hepatitis E virus (HEV) is a long-ignored virus that has spread globally with time. It ranked 6th among the top risk-ranking viruses with high zoonotic spillover potential; thus, considering its viral threats is a pressing priority. The molecular pathophysiology of HEV infection or the underlying cause is limited. Therefore, we incorporated an unbiased, systematic methodology to get insights into the biological heterogeneity associated with the HEV. Our study fetched 93 and 2016 differentially expressed genes (DEGs) from chronic HEV (CHEV) infection in kidney-transplant patients, followed by hub module selection from a weighted gene co-expression network (WGCN). Most of the hub genes identified in this study were associated with interferon (IFN) signaling pathways. Amongst the genes induced by IFNs, the 2′-5′-oligoadenylate synthase 3 (*OAS3*) protein was upregulated. Protein-protein interaction (PPI) modular, functional enrichment, and feed-forward loop (FFL) analyses led to the identification of two key miRNAs, i.e., miR-222-3p and miR-125b-5p, which showed a strong association with the *OAS3* gene and TRAF-type zinc finger domain containing 1 (*TRAFD1*) transcription factor (TF) based on essential centrality measures. Further experimental studies are required to substantiate the significance of these FFL-associated genes and miRNAs with their respective functions in CHEV. To our knowledge, it is the first time that miR-222-3p has been described as a reference miRNA for use in CHEV sample analyses. In conclusion, our study has enlightened a few budding targets of HEV, which might help us understand the cellular and molecular pathways dysregulated in HEV through various factors. Thus, providing a novel insight into its pathophysiology and progression dynamics.

## 1. Introduction

Animal-sourced viruses have posed a severe threat to public health globally, as seen by the recent appearance and spread of zoonotic viruses such as the Ebola virus and the Severe Acute Respiratory Syndrome Coronavirus 2 (SARS-CoV-2). The Hepatitis E virus (HEV) ranks among the top 10 risk-ranking viruses with a high potential for zoonotic spillover. Therefore, considering its viral risks is a growing concern [1]. The current status of HEV corresponds to 1/8th of the global population. Among them are the recent and ongoing infections of 15–110 million individuals due to increased prevalence in developed countries [2]. HEV comprises around 7.2 kb quasi-enveloped, positive sense, single-stranded RNA genome comprising three significant open reading frames (ORFs) and the fourth in genotype 1 [3]. It commonly causes acute hepatitis with chronicity in pregnant women, organ transplant patients, and immunocompromised patients [2]. HEV is involved in extrahepatic manifestations and zoonotic transmission among different hepatitis viruses [4,5]. Still, the molecular pathophysiology of HEV infection or the underlying cause is limited, and no effective cure or hepatitis E vaccine is available globally [6]. 

A virus can successfully infect a host by breaching numerous levels of its defense mechanism and effectively using host factors for its replication [7]. Lately, microRNAs (miRNAs), one of the host factors, have come into the spotlight due to their significant role in regulating viral infections at the post-transcriptional level. They are small RNAs (non-coding) that are primarily engaged in suppressing gene expression of either cellular or viral RNAs amid infection [8]. Few studies have acknowledged the function of miRNAs in stimulating gene expression in non-viral diseases [9]. The miRNAs exhibit pleiotropy, which indicates that they have several messenger RNA (mRNA) targets and vice versa. Multiple miRNAs’ target sites are sometimes co-localized at a single gene, synergistically repressing the mRNA [10,11]. Different studies report that the functions of several miRNAs are cell- or organ-specific. Through various target sequences, a particular kind of miRNA can influence multiple interacting cellular pathways and thus play a crucial part in developing diseased conditions.

In contrast to miRNA, which regulates genes post-transcriptionally, transcription factors (TFs) are the ones that regulate the genes at the transcriptional level [12]. In addition, miRNAs and TFs not only regulate the genes but also regulate each other. Intricately intertwined miRNA and TF interactions result in feed-forward loops (FFLs) or feedback loops (FBLs), in which a TF modulates a miRNA, or a miRNA negatively regulates a TF, and the two of them co-regulate a common target. Three types of FFLs can be categorized according to their master regulators: TF-FFL, miRNA-FFL, and composite FFL [13]. 

We incorporated an unbiased, systematic methodology to get insights into the biological heterogeneity associated with the HEV. Utilizing microarrays allows researchers to swiftly investigate the expression of numerous genes in a reaction. In addition, it allows one to choose the dataset based on their research design. Certain limitations persist in comprehensively exploring the entire biological system for any disorder. Weighted gene co-expression network analysis (WGCNA) is a powerful and effective algorithm attempting to dig valuable information from gene expression profiles by building gene modules and trying to justify the significance of gene modules from a biological point of view [14,15,16,17]. FFLs can engineer recurrent network motifs to enhance the robustness of gene regulation in mammalian genomes [18]. A three-node FFL can be extended to form a four-node or five-node FFL by adding further interaction links. Three-node miRNA FFL comprises a TF, a miRNA, and a common gene target, with miRNAs dominating or driving the whole network [13]. Previous studies have shown that miRNAs may be therapeutic in treating viruses such as Herpes Simplex Virus (HSV), Dengue, Human Immunodeficiency Virus-1 (HIV-1), Hepatitis C virus (HCV), and influenza [19,20]. As they meet all the requirements, including specificity, sensitivity, and accessibility, thus miRNAs can also become excellent biomarkers [21]. Therefore, identifying miRNA networks will take us one step closer to the advancement in diagnostic and therapeutic purposes related to HEV. 

HEV-associated mRNA expression datasets corresponding to two groups, mainly whole blood (WB) and monocytes (Mo), were extracted from National Center for Biotechnology Information (NCBI)-Gene Expression Omnibus (GEO). Differentially expressed genes (DEGs) were identified in both groups, followed by the establishment of a weighted gene co-Expression network (WGCN). Hub module selection was conducted after DEGs were individually passed as input to WGCNA. Thirteen overlapping hub genes were shortlisted by functional enrichment and protein-protein interaction (PPI) analysis. Further analysis of these genes using three-node miRNA FFL revealed the key TF, miRNA, and gene that are associated with the development of HEV. Our study has enlightened a few budding targets of HEV, which could serve as an effective tool against the zoonotic HEV.

## 2. Materials and Methods 

### 2.1. HEV mRNA Expression Profile Extraction and Differential Expression Analysis (DEA)

We accessed the NCBI-GEO [22] (https://www.ncbi.nlm.nih.gov/geo/, accessed on 1 August 2022) utilizing “HEV” and “Hepatitis E Virus” as appropriate keywords for HEV-associated mRNA expression profile retrieval. The search results were further trimmed down in accordance with the following inclusion criteria: (1) the dataset(s) must be “expression profiling by array” type along with HEV infection being present in “Homo Sapiens” as host; (2) the dataset must include raw and processed microarray data; (3) the dataset must have control and infected sample types; (4) the submission date of the dataset must be within last ten years (i.e., 2012 to 2022); (5) the dataset must contain greater than 25 samples. We excluded studies devoid of case reports, review articles, abstracts, non-human samples, and cell-line-based experimental study designs. The series matrix expression file of the selected dataset was retrieved from GEO, followed by quality checks. ARSyNseq function (with unknown batch settings) was available within NOISeq to acquire batch-corrected expression values. Mapping of probe IDs to their corresponding HUGO Gene Nomenclature Committee (HGNC) symbols was handled via GEO2R. Averaging expression values for those gene(s) mapping to several probe IDs was conducted to avoid redundancy. *p*-values and $\log_{2}\left(\mathrm{fold}\;\mathrm{change}\right)$ values of all genes across two sample groups were computed utilizing limma [23]. The genes corresponding to a *p*-value <0.05 and $\left|\log_{2}\left(\mathrm{fold}\;\mathrm{change}\right)\right|>0.1$ were considered as differentially expressed across two sample groups. HGNC multi-symbol checker (https://www.genenames.org/tools/multi-symbol-checker/, accessed on 5 August 2022) was queried thereafter to obtain DEGs with officially approved HGNC symbols.

### 2.2. HEV-Specific WGCN Construction and Hub Module Selection

WGCN formation and module(s) selection are discussed in detail in [24]. Module eigengene (ME) and MEdissimilarity (MEdiss) were calculated. ME dendrogram was examined based on Pearson correlation to merge module(s) with comparable high expression profiles. Standard intramodular connectivity (k.in) and module membership (MM) were computed for every module. The module(s) with a significantly higher correlation between k.in and MM were regarded as the hub module(s). 

### 2.3. PPI Network Construction and Modular Analysis

The hub module DEGs of both Mo and WB groups were collectively given as input to Search Tool for the Retrieval of Interacting Genes (STRING, https://string-db.org/, accessed on 21 August 2022) v11.5 web-based tool [25] in order to establish a PPI network corresponding to highest confidence (i.e., interaction score >0.9) and subsequently visualized utilizing Cytoscape v3.91 [26]. The parameters used for PPI hub module selection are discussed in detail in [27].

### 2.4. Pathway and Gene Ontology (GO) Term Enrichment Analyses for Hub Gene Selection

All the PPI hub module genes were given as input to the Enrichr web server [28] (https://maayanlab.cloud/Enrichr/, accessed on 25 August 2022), and the top 10 significantly (*p*-value < 0.05) enriched pathways and GO terms were collected from BioPlanet, GO-Biological Process (BP), GO-Molecular Function (MF), and GO-Cellular Compartment (CC) libraries. The genes overlapping between pathway, GO-BP, GO-MF, and GO-CC genesets were regarded as our hub genes.

### 2.5. HEV-Specific Three-Node miRNA FFL Construction and Analysis

ChEA v3.0 database [29] was queried in order to fetch significant [corresponding to score (*p*-value) <0.05] human TFs regulating our hub mRNAs. miRNAs repressing our hub mRNAs and TFs (compiled from ChEA) were retrieved from miRWalk v3.0 [30] (miRNAs with score >0.95 and binding only on $3^{\prime }\;\mathrm{UTR}$ region only), StarBase v2.0 [31], and miRTargetLink v2.0 [32] (strongly validated miRNAs) databases to form miRNA-TF and miRNA-mRNA pairs. A union of miRNAs from all these databases was taken in order to encompass a wide spectrum of miRNAs. These miRNAs were validated via literature, and only HEV-associated miRNAs were retained for further analysis. To establish a closed three-node miRNA FFL, all three interaction pairs (i.e., TF-mRNA, miRNA-mRNA, and miRNA-TF) were altered in such a manner that the FFL is composed of only overlapping miRNAs, TFs, and mRNAs. The miRNA FFL was thereafter visualized using Cytoscape.

### 2.6. Identification of Secondary Structures and Protein-Protein Docking Studies

The α-fold structure of human $2^{\prime }-5^{\prime }$ oligoadenylate synthase (OAS3) protein was extracted from Research Collaborators for Structural Bioinformatics Protein Data Bank (RCSB PDB) database (ID: AF_AFQ9Y6K5F1; residues 1–1087) with global pLDDT (model confidence score) of 86.82 (Figure S1) [33,34]. Only the third domain of OAS3 (referred to as OAS3_D3), having the three aspartic acid residues Asp816, Asp818, and Asp888, which corresponds to the catalytic triad in human OAS1, are conserved (Figure S5) [35]. Therefore, the third domain of *OAS3* (residues 743–1087) was selected for protein-protein docking studies. The NMR-determined structure of the TRAF-type zinc finger domain containing 1*TRAFD1* or *FLN29* genes was downloaded from the RCSB PDB database (PDB ID: 2D9K; residues 1–75) (Figure S5). Through the CASTp server v3.0 (http://sts.bioe.uic.edu/castp/, accessed on 30 August 2022), binding site residues for TRAFD1 were retrieved and used as constraints for protein docking via the HADDOCK v2.4 web server [de Vries et al., 2010] (http://wenmr.science.uu.nl/haddock2, accessed on 2 September 2022). In addition, the best-docked complexes with the lowest intermolecular energies were selected for further study [van Dijk and Bonvin, 2006; Dominguez et al., 2007]. Binding free energy calculation (in kcal/mol) of the docked complex was predicted by the PRODIGY web server [36] (https://wenmr.science.uu.nl/prodigy/, accessed on 5 September 2022). The final docked complex was analyzed utilizing Protein Interactions Calculator (PIC) to confirm the interactive residues within the hydrogen-bond distance (3.5 Å) [37]. The structure and docked complex with their interacting residues were visualized using PyMol [38]. The inhibition constant ($K_{i}$) for the docked complex were estimated by utilizing the formula:

$$K_{i}=\exp\left[\left(\Delta G\right)∕\left(R*T\right)\right]$$

where, R (universal gas constant) is $1.985\times 10^{-3}{\;\mathrm{kcal}\;\mathrm{mol}}^{-1}\;K^{-1}$, ΔG is docking energy, and T (temperature) is 298.15 K [39].

## 3. Results

### 3.1. HEV mRNA Expression Profile Extraction and DEA

**Figure 1:**
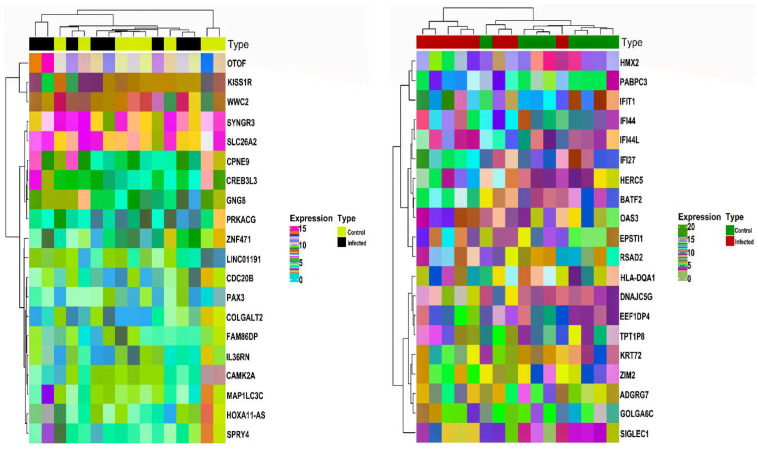
Annotation heatmap displays the distribution (expression) of the top 10 down and upregulated DEGs in the case of Mo (**left panel**) and WB (**right panel**). The cluster dendrograms signifying the Euclidean distance-based hierarchical clustering for both rows and columns are shown along the left and top sides of the plot. Sample type annotation bar is shown at the top of the heatmap. DEGs, Differentially Expressed Genes; Mo, Monocytes; WB, Whole Blood.

Based on the abovementioned inclusion and exclusion criteria, we selected the HEV mRNA expression profile with accession number GSE36539. The dataset comprised 16 WB patient samples (i.e., eight WB control and eight WB infected) and 16 Mo patient samples (eight Mo control and eight Mo infected). For post-batch correction, we bifurcated the two cell types’ data, with each set comprising 16 samples. After averaging expression values corresponding to duplicate genes, a total of 15991 and 15990 unique gene symbols were retained for Mo and WB sample groups. Corresponding to a *p*-value <0.05 and $\left|\log_{2}\left(\mathrm{fold}\;\mathrm{change}\right)\right|>0.1$, we obtained 190 and 2749 DEGs corresponding to Mo and WB sample groups. After checking via the HGNC multi-symbol checker and filtering by Pigengene, we were finally left with 93 and 2016 DEGs corresponding to Mo and WB sample groups. Figure 1 shows the expression heatmap plot of the top 10 down and upregulated DEGs in the case of Mo and WB groups. The sample annotation bars are placed at the top of the heatmap. The most highly up and downregulated DEGs were *OTOF* [$\log_{2}\left(\mathrm{fold}\;\mathrm{change}\right)=1.25$] and *HOXA11-AS* [$\log_{2}\left(\mathrm{fold}\;\mathrm{change}\right)=-1.05$] in the case of Mo while *RSAD2* [$\log_{2}\left(\mathrm{fold}\;\mathrm{change}\right)=4.04$] and *GOLGA6C* [$\log_{2}\left(\mathrm{fold}\;\mathrm{change}\right)=-1.94$] in the case of WB. 

### 3.2. HEV-Specific WGCN Construction and Hub Module Selection

**Figure 2:**
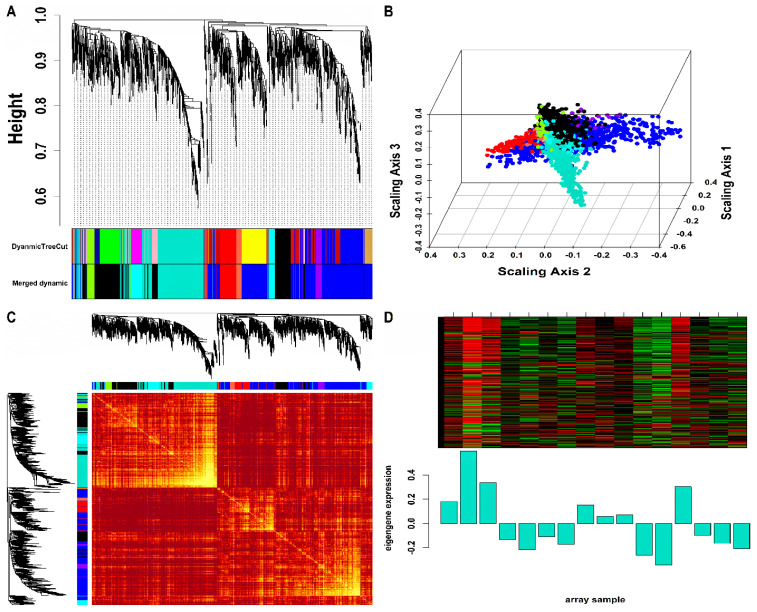
(**A**) Hierarchical clustering dendrogram of 2016 WB-DEGs clustered based on dissTOM together with original (16) and merged (9) module colors. The sizes of the original modules were as follows: black (108), blue (300), brown (207), cyan (42), green (151), green-yellow (63), grey (6), magenta (85), midnight-blue (39), pink (94), purple (65), red (126), salmon (43), tan (51), turquoise (455), yellow (181). In addition, the sizes of merged modules were as follows: black (392), blue (739), cyan (127), green-yellow (63), grey (6), purple (65), red (126), salmon (43), turquoise (455). (**B**) 3D MDS plot where each colored point denotes a gene belonging to the module of its equivalent color in the case of the WB group. (**C**) Representation of WGCN as a TOM plot for WB-DEGs. Genes within columns and their corresponding rows were hierarchically clustered by cluster dendrograms (displayed along the top and left side of the plot). Progressively darker and lighter red colors within the matrix indicate higher and lower topological overlap among genes. Dark-colored blocks along the diagonal signify the modules. (**D**) Expression heatmap of turquoise module genes where the rows and columns correspond to genes and samples. The red and green color bands within the heatmap imply higher and lower expression levels across turquoise module genes. In addition, the corresponding ME expression levels (along the *y*-axis) across all samples (along the *x*-axis) are displayed at the bottom panel of the module heatmap in the form of a barplot. WGCN, Weighted Gene Co-expression Network; TOM, Topological Overlap Matrix.

**Figure 3:**
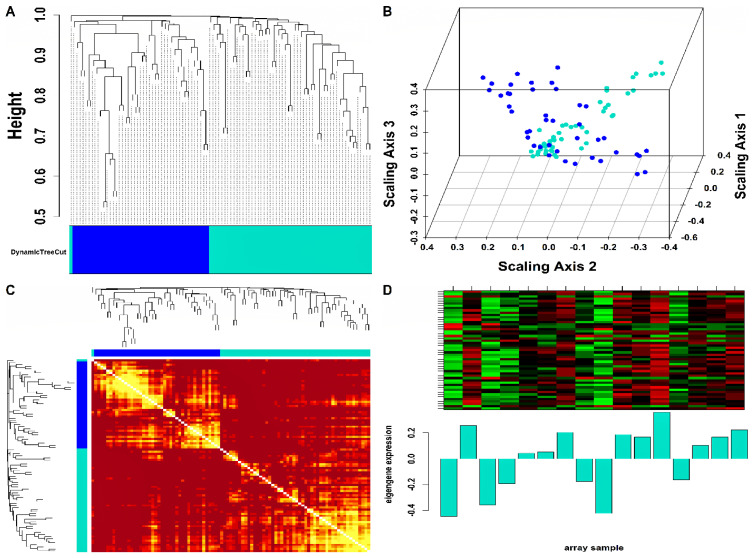
(**A**) Hierarchical clustering dendrogram of 93 Mo-DEGs clustered based on dissTOM and two color-coded modules obtained using Dynamic Tree Cut. The sizes of the module were as follows: blue (42) and turquoise (51). (**B**) 3D MDS plot where each colored point denotes a gene belonging to the module of its equivalent color in the case of the Mo group. (**C**) Representation of WGCN as a TOM plot for Mo-DEGs. Genes within columns and their corresponding rows were hierarchically clustered by cluster dendrograms (displayed along the top and left side of the plot). Progressively darker and lighter red colors within the matrix indicate higher and lower topological overlap among genes. Dark-colored blocks along the diagonal signify the modules. (**D**) Expression heatmap of turquoise module genes where the rows and columns correspond to genes and samples. The red and green color bands within the heatmap imply higher and lower expression levels across turquoise module genes. In addition, the corresponding ME expression levels (along the *y*-axis) across all samples (along the *x*-axis) are displayed at the bottom panel of the module heatmap in the form of a barplot.

Mo-associated (93) and WB-associated (2016) HEV DEGs were passed as input to WGCNA. The WGCN was generated at β=15 (corresponding to $R^{2}=0.89$) and β=9 (corresponding to $R^{2}=0.8$) for Mo and WB sample groups, respectively. The clustering tree (hierarchical) and dynamic tree cut algorithm resulted in sixteen (i.e., black, blue, brown, cyan, green, green-yellow, grey, magenta, pink, midnight-blue, purple, red, salmon, tan, turquoise, yellow) and two (blue, turquoise) color-coded modules corresponding to WB and Mo sample groups. The merging of modules with highly co-expressed gene patterns was possible only in the WB group (due to a large number of genes) by cutting the dendrogram at the height of 0.25. After merging, the initial sixteen modules of the WB group were amalgamated into nine color-coded modules (i.e., black, blue, cyan, green-yellow, grey, purple, red, salmon, and turquoise). The Mo group’s two modules were retained due to no possible merging between modules. Since the grey module consists of unassigned genes, we discarded it for further analysis. Figures S2 and S3 show plots for β in consideration with scale-free topology (SFT) criteria for Mo and WB sample groups. The clustering tree (hierarchical) of WB-DEGs based on dissTOM and ME with original (16) and merged (9) color modules is shown in Figure 2A. Figure 3A shows the clustering tree (hierarchical) of Mo-DEGs based on dissTOM with the original two modules. Figure 2B and Figure 3B show a multidimensional scaling (MDS) plot of all modules in three scaling dimensions in WB and Mo. WGCN depicting TOM among WB and Mo modules is shown in Figure 2C and Figure 3C. Based on the most significant correlation between MM and k.in (Tables S1 and S2), the turquoise module was chosen as our hub module in the case of both Mo and WB. Figure 2D and Figure 3D show the heatmap plot of turquoise module genes along with their corresponding ME levels in the case of WB and Mo. A total of 51 and 457 DEGs were present in the hub turquoise module within Mo and WB.

### 3.3. PPI Network Construction and Modular Analysis

**Figure 4:**
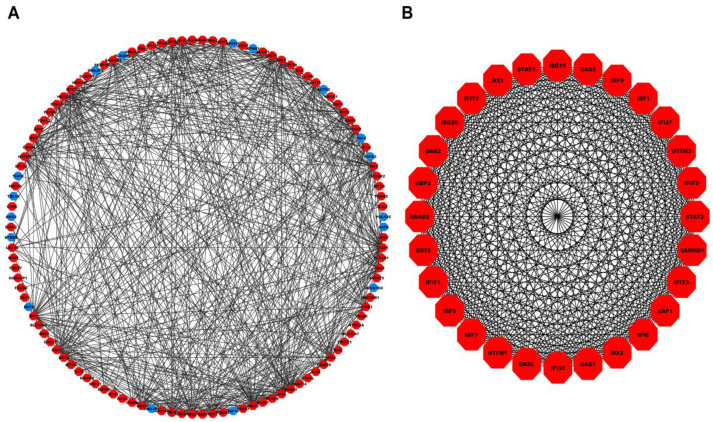
(**A**) PPI network comprising 114 nodes and 545 interaction edges constructed using the STRING database corresponding to interaction score >0.9. The red- and blue-colored nodes signify up and downregulated proteins. (**B**) Top scoring PPI module comprises 28 nodes and 374 edges. PPI, Protein-Protein Interaction.

A total of 506 hub genes within the turquoise module were inputted into the STRING database, of which 477 were mapped to their corresponding proteins. Figure 4A shows the PPI network comprising 114 nodes and 545 edges (corresponding to an interaction score >0.9). Molecular Complex Detection (MCODE) revealed a total of four modules, out of which the first module had the highest score and was considered our PPI hub module. Figure 4B shows the PPI hub module comprising 28 nodes and 374 edges. Essential centrality measures such as node degree, betweenness, closeness, clustering coefficient, topological coefficient, and average shortest path length of the PPI network are shown in Figure S4.

### 3.4. Pathway and GO Term Enrichment Analyses for Hub Gene Selection

A total of 25, 28, 20, and 21 genes within our PPI hub module participated in the top 10 significant pathways, GO-BP, GO-MF, and GO-CC terms, as shown in Tables S3–S6. The most significant pathway, GO-BP, GO-MF, and GO-CC terms were Interferon α/β signaling (*p*-value $=1.89\times 10^{-58}$), cellular response to type I interferon (*p*-value $=2.24\times 10^{-73}$), adenylyltransferase activity (*p*-value $=2.21\times 10^{-8}$), mitochondrial envelope (*p*-value $=2.82\times 10^{-5}$). A total of 13 hub genes overlapped between these top 10 significant pathways, GO-BP, GO-MF, and GO-CC genesets, shown by a Venn plot in Figure S4. The box-and-whisker plots showing the relative expression distribution of these HEV-hub genes are shown in Figure S5.

### 3.5. HEV-Specific Three-Node miRNA FFL Analysis

**Figure 5:**
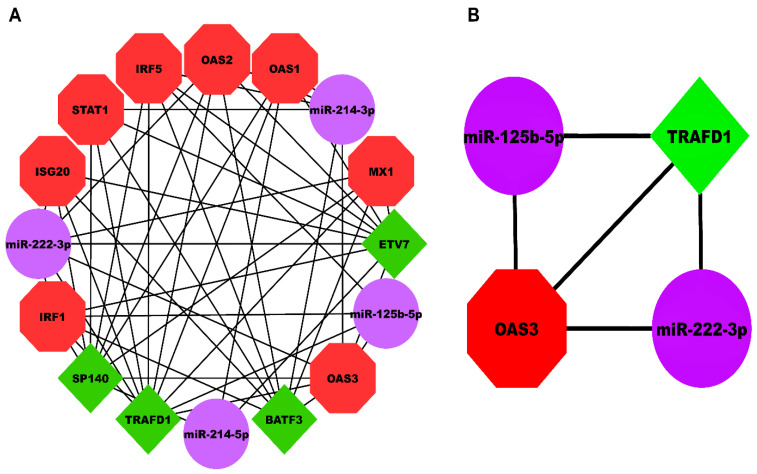
(**A**) HEV-specific three-node miRNA FFL comprising 16 nodes and 51 edges. (**B**) Highest-order subnetwork motif comprising one TF (*TRAFD1*), one miRNA (miR-222-3p), and one mRNA (*OAS3)*. Red-colored octagonal nodes signify hub mRNAs, green-colored diamond nodes signify TFs, and magenta-colored circular nodes signify miRNAs. HEV, Hepatitis E Virus; FFL, Feed-Forward Loop; TF, Transcription Factor; TRAFD1, TRAF-Type Zinc Finger Domain Containing 1; OAS3, 2′-5′-Oligoadenylate Synthase 3.

Our HEV-specific-node miRNA FFL, shown in Figure 5A, comprised 16 nodes and 51 edges. TF-mRNA, miRNA-mRNA, and miRNA-TF pairs embodied 31, 14, and 6 edges among the whole FFL. Amongst all the FFL nodes, 8, 4, and 4 belonged to mRNAs, miRNAs, and TFs. The degree range of miRNAs, mRNAs, and TFs in the FFL varied from 3 to 7, 5 to 7, and 8 to 10, respectively. The average degrees of TFs, mRNAs, and miRNAs were 9.25, 5.625, and 4. The three pairs of regulatory relationships between miRNAs, mRNAs, and TFs were summarized in Table S7. Out of all TFs, both *TRAFD1* and *ETV7* were equally regulated by the highest number of miRNAs (i.e., 2). Among all the hub DEGs, *OAS3* was regulated by the highest number of miRNAs (i.e., 3). Tables S8–S10 show the top three miRNAs, mRNAs, and TFs within FFL ranked based on degree, betweenness, closeness, eigenvector, and radiality. Based on the observations from the table, the highest-order subnetwork motif comprised one TF (*TRAFD1*), two miRNAs (miR-222-3p and miR-125b-5p), and one mRNA (*OAS3*), as shown in Figure 5B.

### 3.6. Protein-Protein Docking Analysis and Interaction Studies

**Figure 6:**
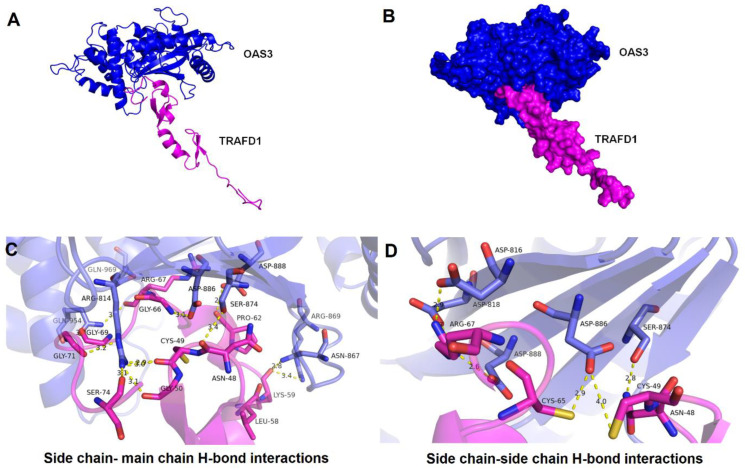
Docked mode of OAS3_D3-TRAFD1 complex and its structural mapping of intramolecular interfaces. (**A**) Cartoon representation of OAS3_D3-TRAFD1 docked complex having low energy score determined by HADDOCK docking. OAS3_D3 is displayed in blue color, and TRAFD1 is represented in magenta color. (**B**) A surface representation of OAS3_D3-TRAFD1 complex. (**C**) Interacting residues between OAS3_D3 and TRAFD1 obtained through hydrogen bonding between the side chain of OAS3_D3 and the main chain of TRAFD1 are shown in licorice representation, OAS3_D3 residues (blue) and TRAFD1 residues (in magenta). (**D**) Interacting residues between OAS3_D3 and TRAFD1 obtained through hydrogen bonding between the side chain of OAS3_D3 and the side chain of TRAFD1 are shown in licorice representation, OAS3_D3 residues (blue) and TRAFD1 residues (in magenta). The atomic distances are shown in yellow dotted lines in angstrom (Å).

The predicted binding residues obtained by CASTp v3.0 corresponding to TRAFD1 protein having solvent accessible surface area $10.735{\;Å}^{2}$ and for OAS3_D3 protein, the catalytic triad- Asp816, Asp818, and Asp888 were further analyzed for docking (Table S11). The nine best clusters generated by docking for the OAS3_D3-TRAFD1 complex are shown in Table S12. The clusters with a low HADDOCK score, low Z-score, and low RMSD values were considered the best-docked complex. The model with a HADDOCK score of −91.7+/−6.1 Kcal/mol, RMSD of 0.7+/−0.5 Å, and Z-score of −2.4 was selected for OAS3_D3-TRAFD1 complex (Figure 6A). The predicted binding affinity (ΔG) and dissociation constant ($K_{d}$) of the OAS3_D3-TRAFD1 complex calculated by the PRODIGY server resulted in values −11.0 kcal/mol and 9.1E-09 M at 25 ℃, respectively. Moreover, the inhibition constant of the OAS3_D3-TRAFD1 complex was computed to be 8.472E-09. Analysis of docked complex (OAS3_D3-TRAFD1) using the PIC server showed the presence of interacting residues showing hydrogen bonding interactions between the side chain of OAS3_D3 and the main chain of TRAFD1 (Table S13a) and between side chains of OAS3_D3 and TRAFD1 (Table S13b). OAS3_D3 is shown to interact with TRAFD1 with buried surface area of $1706.5+/-71.8{\;Å}^{2}$ (Figure 6B). Fourteen amino acid residues of OAS3_D3 are shown to form H-bonds with backbone atoms of TRAFD1 (Figure 6C). Furthermore, seven amino acid residues OAS3_D3 form H-bonds with side chains of TRAFD1 (Figure 6D). Interestingly, among these hydrogen bond interactions, the side chains of catalytic triad residues Asp814, Asp818, and Asp888 of OAS3_D3 jointly interact with side chains of Arg67 of TRAFD1, leading to the formation of salt-bridge-mediated interactions.

## 4. Discussion

We primarily accessed GEO to extract a dataset containing mRNA from WB and Mo samples of kidney transplant patients, each from control and infected. Our analysis identified 93 and 2016 DEGs corresponding to Mo and WB sample groups, which were then rigorously screened using WGCNA to select hub modules containing 51 and 457 DEGs from Mo and WB sample groups, respectively. Furthermore, the PPI network modular analysis indicated four modules, out of which the first module was considered our PPI hub module. Pathway and GO term enrichment analyses of the PPI hub module revealed thirteen overlapping genes between the top 10 significant pathways, GO-BP, MF, and CC genesets. These thirteen upregulated genes—Guanylate BindingProtein 2 (*GBP2*), Interferon Regulatory Factor 1 (*IRF1*), Interferon Regulatory Factor 5 (*IRF5*), Interferon Regulatory Factor 7 (*IRF7*), Interferon Regulatory Factor 9 (*IRF9*), Interferon Stimulating Gene 20 (*ISG20*), MX Dynamin Like GTPase 1 (*MX1*), MX dynamin Like GTPase 2 (*MX2*), 2′-5′-Oligoadenylate Synthase 1 (*OAS1*), 2′-5′-Oligoadenylate Synthase 2 (*OAS2*), *OAS3*, SAM Domain And HD Domain-Containing Protein 1 (*SAMHD1*), and Signal Transducer And Activator Of Transcription 1 (*STAT1*) regarded as the hub genes were obtained. HEV-associated miRNAs and TFs related to these hub genes were also detected. Within our work, we utilized key transcriptional level interaction, i.e., miRNA → gene, miRNA → TF, and TF → gene regulation, for constructing a miRNA-centered FFL, which portrays a critical role in HEV pathogenesis.

A limited number of studies have been conducted concerning miRNA regulation during chronic HEV infection. Based on the centrality measures, we identified miR-222-3p (member of the miR-221/222 family) and miR-125b-5p as the top-ranked miRNAs engaged in the FFL regulation involving *TRAFD1* and *OAS3* in HEV. The miR-222-3p has already been implicated in regulating downstream acute HEV in vitro [40]. A study by Elghoroury et al. predicted the diagnostic value of miR-222 as a potential biomarker in diagnosing liver injuries or progression, cirrhosis, and Hepatocellular Carcinoma (HCC) caused by viral infections [41,42]. A case report conducted by Lin et al. discovered that re-infected chronic HEV patient promotes rapid development of HCC and cirrhosis without Hepatitis B Virus (HBV) [43]. This led to the fact that HEV re-infection might boost the progression of HCC in patients regardless of chronic HBV infection. Thus, showing the significance of miR-222 as a potential biomarker in the case of HEV. In addition, a recent study on the acute and chronic HEV-infected serum showed that the miR-125b-5p plays a pivotal role in the suppression of cell proliferation, replication, and apoptosis in HEV and can act as a biomarker in early detection and differentiation between acute and chronic infection [44] which in turn provide us with another potential miRNA as a candidate for biomarker.

Most of the hub genes detected in our work are associated with interferon (IFNs) signaling pathways. Among the genes induced by IFNs, oligoadenylate synthase (OAS) proteins have been identified as enzymes that mediate antiviral effector functions. In humans, the OAS family proteins OAS1, OAS2, and OAS3 are involved in RNase L activation pathways for the degradation of cellular and viral RNA. Recent reports suggested that the 110 kDa OAS3 protein gets activated at lower RNA concentrations than 33-kDa OAS1 and, on average, synthesizes longer $2^{\prime }-5^{\prime }$-linked oligoadenylates (2-5As) to activate RNase L [35]. This is further supported by the previous studies that established the potential roles of OAS3 p100 in mediating the antiviral activity of the dengue virus [45] and HCV [46] in an RNase L-dependent manner. Interestingly, both Dengue virus and HCV are from the same family, *Flaviviridae,* and could induce hepatitis [47].

In addition to miR-125b-5p and miR-222-3p interaction with the *OAS3*, our network analysis also observed *TRAFD1* (Tumor necrosis factor receptor-associated factor-type zinc finger domain containing 1) as a critical TF implicated in the regulatory aspects of HEV infection. *TRAFD1* expression is inducible by interferon and suppresses Toll-like receptor 4-mediated NF-κB activation by binding to tumor necrosis receptor associated factor 6 (*TRAF6*) [48]. A study undertaken by Green et al. performed *OAS1b*-dependent immune transcriptional profiles of West Nile Virus (WNV) infection, which resulted in the hypothesis that *TRAFD1* may contribute to innate immune protection mediated by the *OAS1b* network [49]. In lung adenocarcinoma (LUAD), IFN-γ response genes, *TRAFD1* is reported to exhibit significant expression and a strong positive correlation with *OAS3* [50]. As it has demonstrated relevance in non-viral diseases, it would be interesting to untangle its effects on viral infections, especially HEV. Since HEV infection in organ-transplanted patients can trigger the onset of immune responses, *TRAFD1* can thus act as a master regulator to control the excessive innate immune response [51].

Our first hypothesis revolves around the association of miR-125b-3p, miR-222-3p, and *OAS3*. The miRNAs finalized based on the obtained results have a significant role in HEV, as described by the previous studies. It has been shown that miR-125b-3p upregulates in the case of chronic HEV infection, whereas miR-222 downregulates [40] by enhancing the *OAS3* expression in HEV. In addition, the upregulation of *OAS3* was observed and associated with miR-125b-5p, miR-222-3p, and *TRAFD1* in our study. As miR-125b-5p and *OAS3* are upregulated, we can hypothesize that miR-125b-5p positively regulates the *OAS3* (having antiviral properties). This hypothesis was supported by an earlier study demonstrating that miRNAs can influence viral pathogenesis by either directly modifying viral gene expression or promoting cellular antiviral responses [52]. There is a strong possibility of miR-222-3p showing positive regulation with *OAS3* as well, which is supported by the study showing miR-222 downregulation by enhancing the target gene in HEV [40], which needs further validation through in vitro and in vivo analysis.

Another hypothesis revolves around the connection of *TRAFD1* with *OAS3*. *TRAFD1* shows a negative feedback regulation related to excessive immune response [48]. It thus may be involved in the possible inhibition of upregulated *OAS3* as *OAS3* is an interferon stimulating gene (*ISG*) associated with innate immunity. To verify the above hypothesis, we conducted protein-protein docking where the inhibition constant (K_i_) with 8.472E-09 value and dissociation constant (K_d_) with −11.0 kcal/mol values showed a strong inverse association between TRAFD1 and OAS3 protein.

Our third hypothesis connects the miR-125b-3p and miR-222-3p with *TRAFD1*. Here as the miRNAs are showing a positive regulation with *OAS3*, there is a possibility that miRNAs are involved in inhibiting the *TRAFD1* as *TRAFD1* negatively regulates *OAS3*. The two miRNAs can work in combination or individually to affect *OAS3* and *TRAFD1*.

Based on the above hypotheses, we can conclude that miR-125b-3p and miR-222-3p positively regulate the *OAS3* and negatively regulates *TRAFD1* resulting in higher expression of *OAS3* against HEV. Although to prove this hypothesis, further validation is required through in vitro studies.

## 5. Conclusions

This study consigns the initial report of miRNA signatures determined in WB and Mo samples of kidney-transplanted chronic hepatitis E virus-infected patients. To our knowledge, it is the first time that miR-222-3p has been described as a reference miRNA for use in CHEV sample analyses. The three-node miR-222-3p/miR-125b-5p-*OAS3*-*TRAFD1* regulatory network provides a novel insight into understanding the molecular mechanism of chronic HEV. Further experimental studies are needed to confirm the importance of their role in CHEV infection.

## Data Availability

The dataset used in our work is available in NCBI-GEO at https://www.ncbi.nlm.nih.gov/geo/query/acc.cgi?acc=GSE36539, accessed on 1 August 2022, and can be accessed with GSE36539.

## Supplementary Materials

The following supporting information can be downloaded at: https://www.mdpi.com/article/10.3390/genes14010042/s1, Figure S1: Three-dimensional model of human OAS3 and TRAFD1 proteins and their respective active site binding pocket residues; Figure S2: Quality check plots to assess value of β with respect to scale-free topology (SFT) criteria for Monocytes (Mo) DEGs; Figure S3: Quality check plots to assess value of β with respect to scale-free topology (SFT) criteria for Whole Blood (WB) DEGs; Table S1: Module membership (MM) vs intramodular connectivity (k.in) correlation and p-values for Monocytes (Mo) modules; Table S2: Module membership (MM) vs intramodular connectivity (k.in) correlation and p-values for Whole Blood (WB) modules; Figure S4: Centrality measures showing node degree distribution, betweenness, closeness, clustering coefficient, average shortest path length, neighborhood connectivity, topological coefficient, and radiality of PPI network; Table S3: Top 10 significant pathways (based on p-values) and their gene counts associated with PPI hub module genes; Table S4: Top 10 significant GO-BP terms (based on p-values) and their gene counts associated with PPI hub module genes; Table S5: Top 10 significant GO-MF terms (based on p-values) and their gene counts associated with PPI hub module genes; Table S6: Top 10 significant GO-CC terms (based on p-values) and their gene counts associated with PPI hub module genes; Figure S5: Common 13 HEV-hub genes between top 10 significant GO term and pathway genesets and their relative expression distribution shown by box-and-whisker plots across control and infected patient Whole Blood (WB) samples; Table S7: Summary of regulatory relationships between HEV-associated mRNAs, miRNAs, and TFs; Table S8: Top 3 miRNAs ranked based on centrality measures such as degree, betweenness, closeness, radiality, and eigenvector; Table S9: Top 3 mRNAs ranked based on centrality measures such as degree, betweenness, closeness, radiality, and eigenvector; Table S10: Top 3 TFs ranked based on centrality measures such as degree, betweenness, closeness, radiality, and eigenvector; Table S11: Active residues of domain 3 of OAS3 (AF_Q9Y6K5-F1) and FLN29 or TRAFD1 (PDB ID: 2D9K) proteins identified using literature and predicted using CASTp web server respectively; Table S12: Statistical analysis for HADDOCK generated OAS3-TRAFD1 docked complexes; Table S13: Interfacial residues in docked complex of OAS3-TRAFD1. Protein-protein main chain/side chain-side chain hydrogen bonds. Chain A represent OAS3_D3 protein and chain B represent TRAFD1 protein.
