# Supplementary material for: Integrative Multiomics and Regulatory Network Analyses Uncovers the Role of OAS3, TRAFD1, miR-222-3p, and miR-125b-5p in Hepatitis E Virus Infection
Source: Genes (Basel). 2022 Dec 23;14(1):42. doi: 10.3390/genes14010042 (PMC9859139; doi:10.3390/genes14010042)
Supplement: Supplementary file 1 [file genes-14-00042-s001.zip › genes-2012677-supplementary.pdf]

Supplementary Data

Materials and methods

Identification of secondary structures and protein-protein docking studies

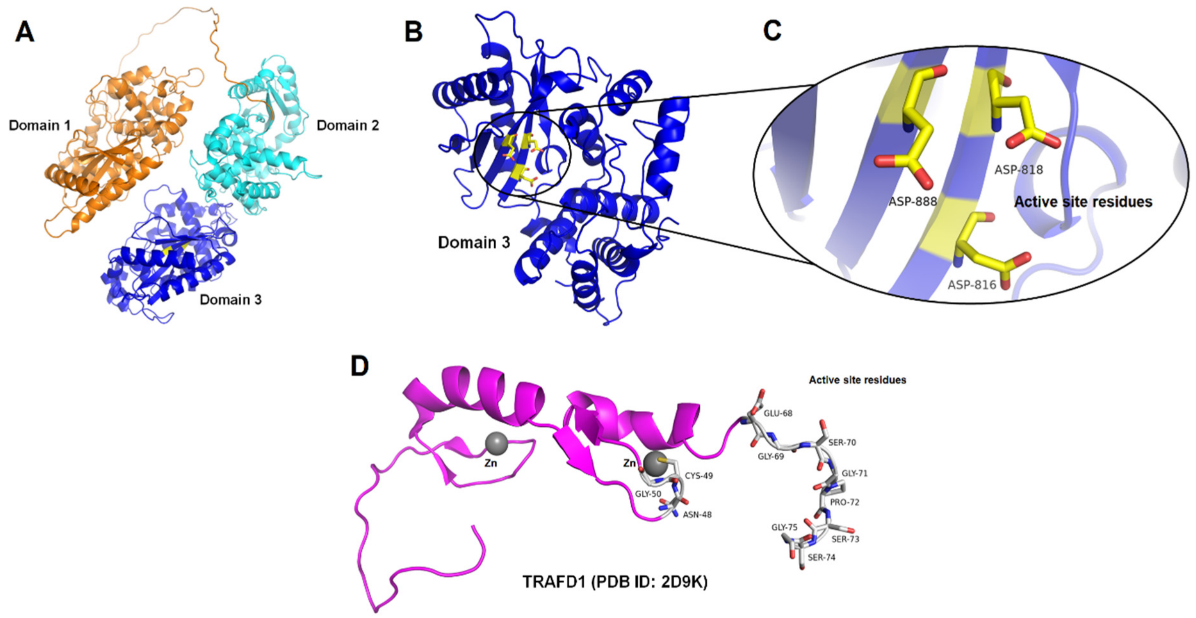

Figure S1. Three-dimensional model of human OAS3 and TRAFD1 proteins and their respective active site binding pocket residues. **(A)** Cartoon representation of human OAS3 protein, domain 1 (D1) in orange color, domain 2 (D2) in cyan color, and domain 3 (D3) in blue color. **(B)** 3-D structure predicted by alpha fold of OAS3_D3 protein represent in blue color and its active site residues. **(C)** The magnified figure of catalytic residues of Asp816, Asp818, and Asp888 of OAS3_D3 protein highlighted in yellow color. **(D)** The NMR solution structure of TRAFD1 (PDB ID: 2D9K) in magenta color, having two zinc ions (in dark grey color) and its predicted active site residues (Cys49, Gly50, Arg51, Glu68, Gly69, Ser70, Gly71, Pro72, Ser73, Ser74, and Gly75) represented in CPK.

Results

HEV-specific WGCN construction and hub module selection

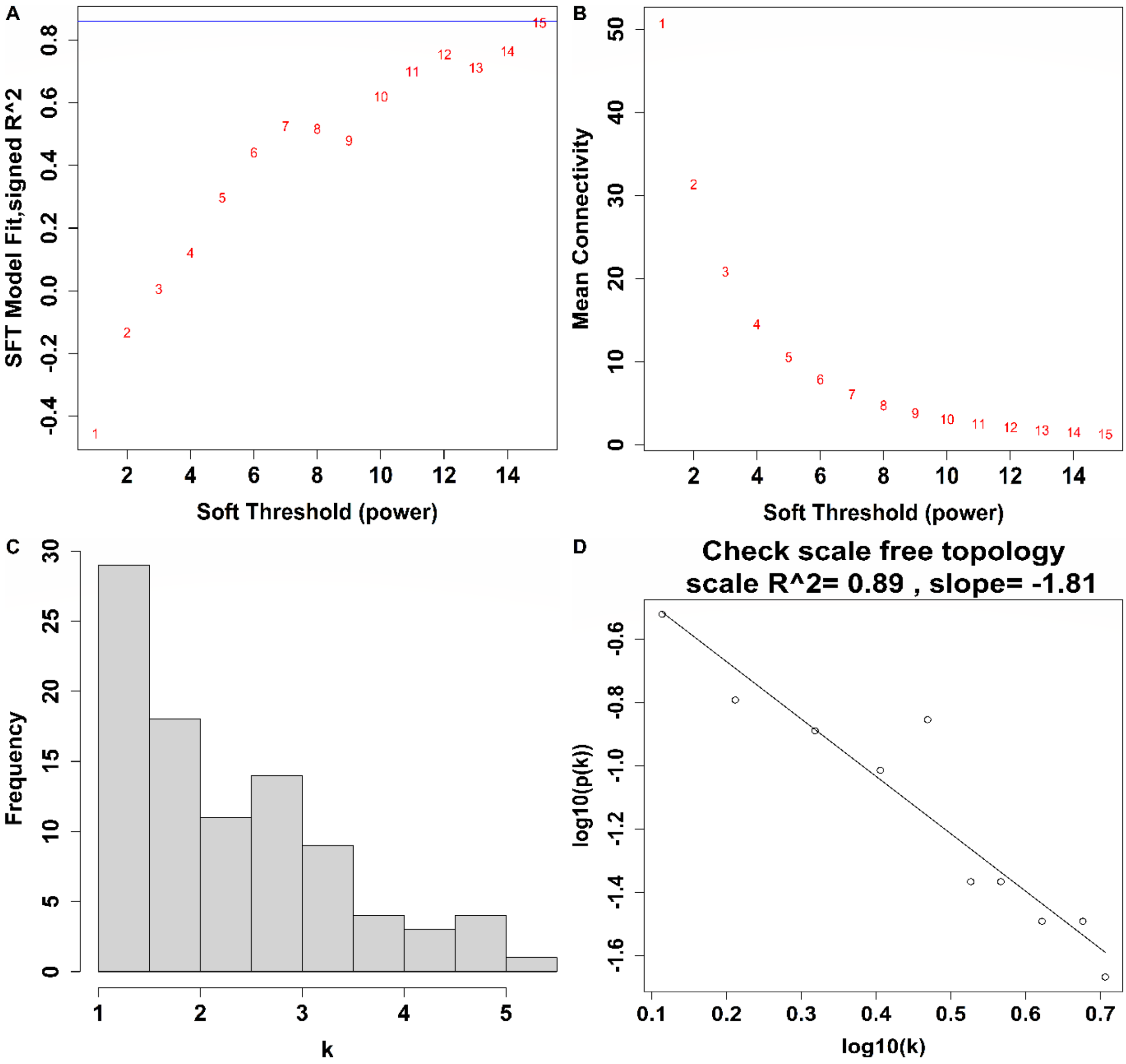

Figure S2. (A) Analysis of scale-free fitting indices (R^2) for various possible β values. **(B)** Analysis of mean connectivity for various possible β values. **(C)** Histogram of network connectivity distribution when $\beta = 15$. **[D]** $log_{10}(k)$ vs $log_{10}(p(k))$ plot of the same histogram where the SFT is depicted by the approximate straight-line relationship (high $R^2 = 0.89$) and a negative value of slope ($slope = -1.81$).

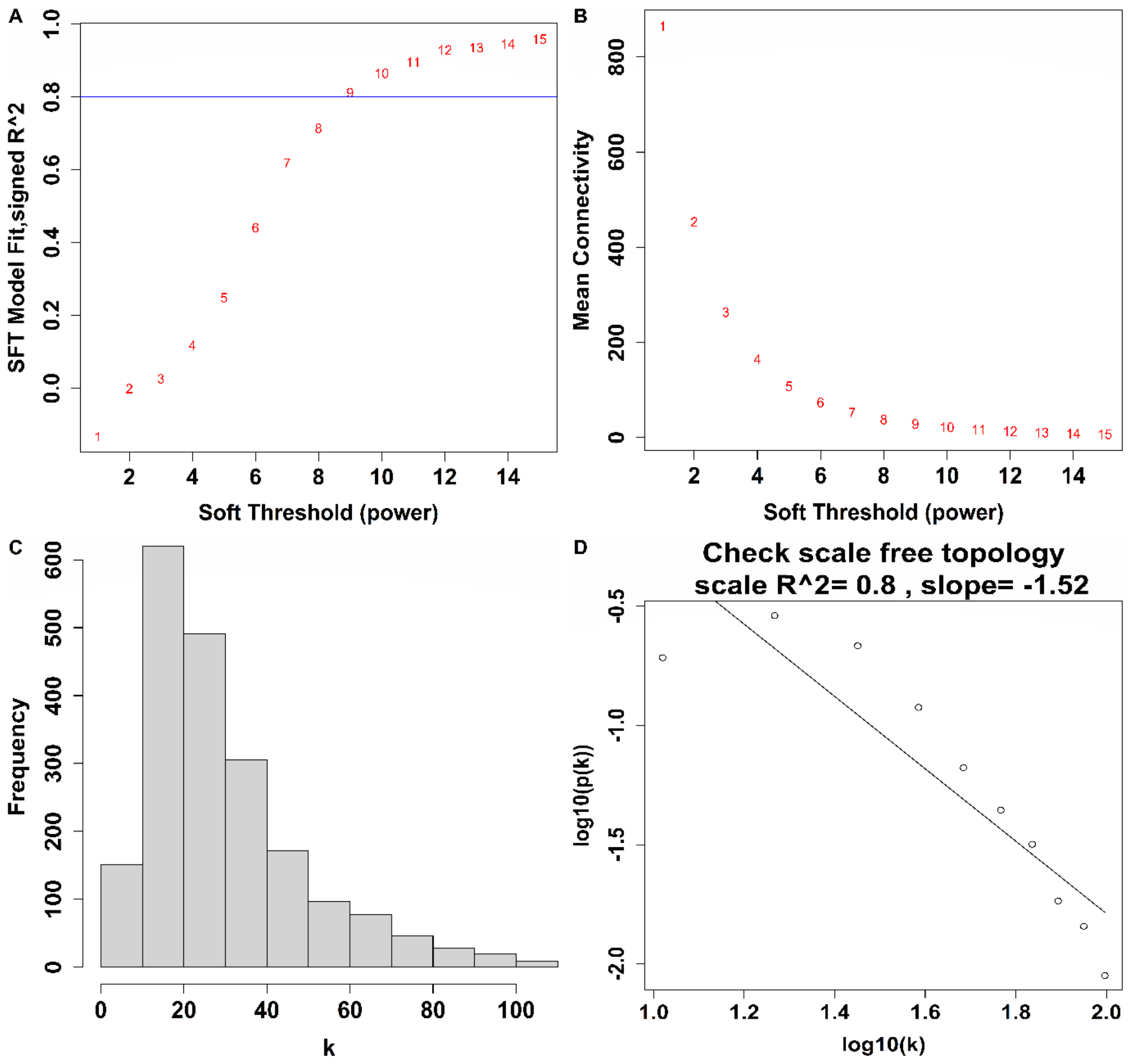

Figure S3. **(A)** Analysis of scale-free fitting indices ($\boldsymbol{R^2}$) for various possible $\boldsymbol{\beta}$ values. **(B)** Analysis of mean connectivity for various possible $\boldsymbol{\beta}$ values. **(C)** Histogram of network connectivity distribution when $\boldsymbol{\beta = 9}$. **[D]** $\boldsymbol{log_{10}(k)}$ vs $\boldsymbol{log_{10}(p(k))}$ plot of the same histogram where the SFT is depicted by the approximate straight-line relationship (high $\boldsymbol{R^2 = 0.8}$) and a negative value of slope ($\boldsymbol{slope = -1.52}$).

Table S1. MM vs k.in correlation and p-values for Mo group modules.

Module	No. of genes	MM vs k.in (correlation)	MM vs k.in (p-value)
**Blue**	42	0.78	1.1×10^{-9}
**Turquoise**	51	0.89	2.5×10^{-18}

Table S2. MM vs k.in correlation and p-values for WB group modules.

Module	No. of genes	MM vs k.in (correlation)	MM vs k.in (p-value)
**Blue**	739	0.7	7.3×10^{-110}
**Turquoise**	455	0.91	2.5×10^{-175}
**Black**	392	0.17	7.3×10^{-4}
**Cyan**	127	0.11	2.2×10^{-1}
**Greenyellow**	63	-0.21	9.9×10^{-2}
**Purple**	65	-0.59	2.3×10^{-7}
**Red**	126	-0.37	2×10^{-5}
**Salmon**	43	0.16	3.1×10^{-1}

PPI network construction and modular analysis

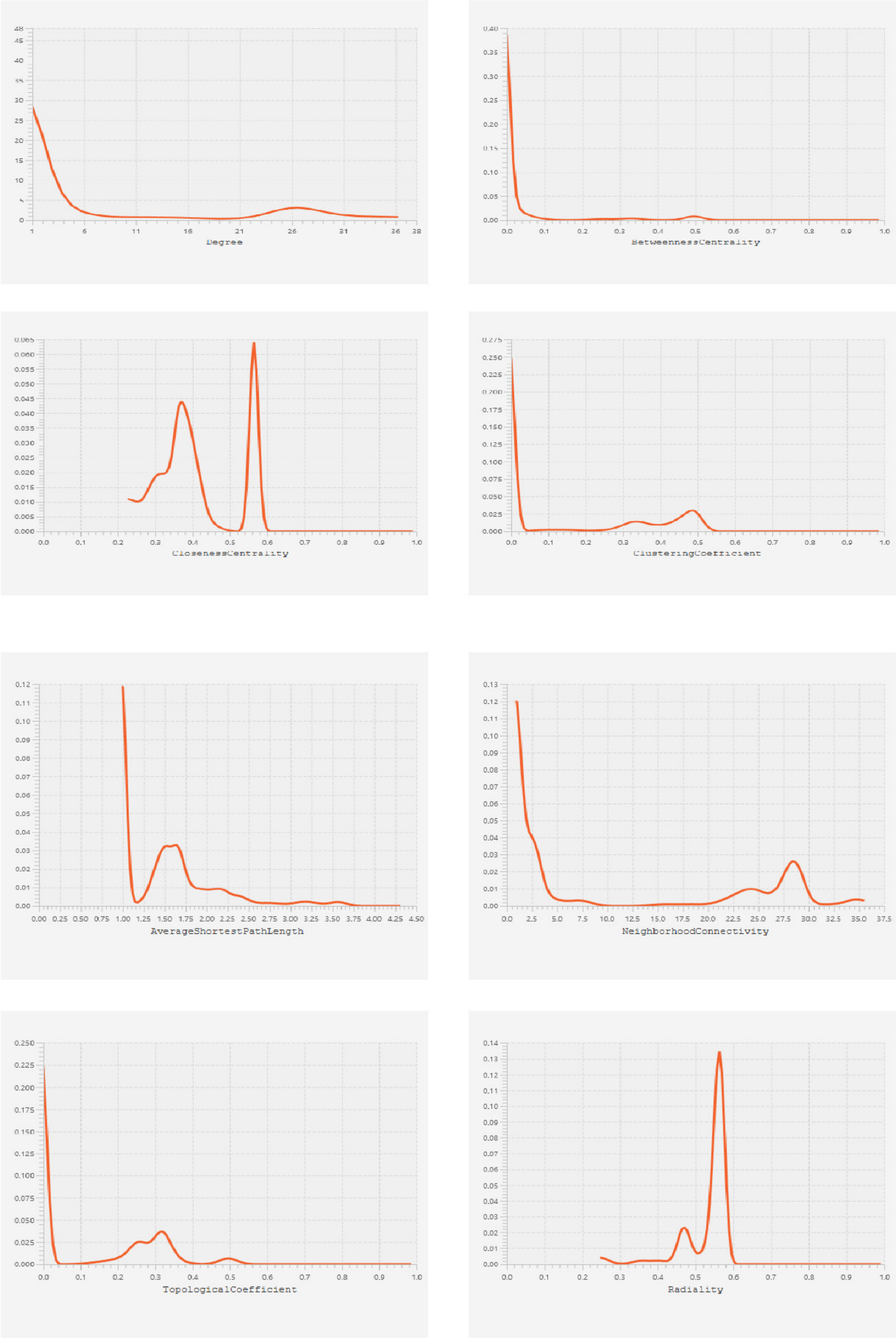

Figure S4. Centrality measures showing node degree distribution, betweenness, closeness, clustering coefficient, average shortest path length, neighborhood connectivity, topological coefficient, and radiality of PPI network.

Pathway and GO term enrichment analyses for hub gene selection

Table S3. Top 10 significant pathways (based on p-values) and their gene counts associated with PPI hub module genes.

Pathways	p-value	Gene Count
Interferon α/β signaling	1.89×10^{-58}	24
Interferon signaling	5.45×10^{-47}	24
Immune system signaling by interferons, interleukins, prolactin, and growth hormones	2.29×10^{-41}	24
Immune system	7.35×10^{-28}	24
Interferon-γ signaling pathway	5.45×10^{-17}	10
Type II interferon signaling (interferon- γ)	2.53×10^{-15}	08
Antiviral mechanism by interferon-stimulated genes	4.19×10^{-08}	05
Interferon- α signaling pathway	2.93×10^{-07}	03
Interferon- β enhancer pathway	9.73×10^{-06}	03
Interleukin-2 signaling pathway	1.46×10^{-05}	08

Table S4. Top 10 significant GO-BP terms (based on p-values) and their gene counts associated with PPI hub module genes.

GO-BP Term	p-value	Gene Count
cellular response to type I interferon (GO:0071357)	2.24×10^{-73}	28
type I interferon signaling pathway (GO:0060337)	2.24×10^{-73}	28
defense response to symbiont (GO:0140546)	1.84×10^{-47}	23
defense response to virus (GO:0051607)	1.08×10^{-46}	23
cytokine-mediated signaling pathway (GO:0019221)	3.28×10^{-43}	28
negative regulation of viral process (GO:0048525)	3.99×10^{-28}	14
negative regulation of viral genome replication (GO:0045071)	3.07×10^{-27}	13
regulation of viral genome replication (GO:0045069)	6.97×10^{-26}	13
interferon-gamma-mediated signaling pathway (GO:0060333)	1.29×10^{-18}	10
cellular response to interferon-γ (GO:0071346)	5.38×10^{-16}	10

Table S5. Top 10 significant GO-MF terms (based on p-values) and their gene counts associated with PPI hub module genes.

GO-MF Term	p-value	Gene Count
adenylyltransferase activity (GO:0070566)	2.21×10^{-08}	04
double-stranded RNA binding (GO:0003725)	3.13×10^{-06}	04
RNA binding (GO:0003723)	1.16×10^{-05}	10
DNA binding (GO:0003677)	9.85×10^{-05}	07
purine ribonucleoside triphosphate binding (GO:0035639)	4×10^{-04}	05
double-stranded DNA binding (GO:0003690)	1.89×10^{-03}	05
nuclease activity (GO:0004518)	3.18×10^{-03}	02
GTPase activity (GO:0003924)	3.33×10^{-03}	03
RNA polymerase II cis-regulatory region sequence-specific DNA binding (GO:0000978)	4.48×10^{-03}	06
cis-regulatory region sequence-specific DNA binding (GO:0000987)	4.48×10^{-03}	06

Table S6. Top 10 significant GO-CC terms (based on p-values) and their gene counts associated with PPI hub module genes.

GO-CC Term	p-value	Gene Count
mitochondrial envelope (GO:0005740)	2.82×10^{-05}	04
mitochondrial membrane (GO:0031966)	4.37×10^{-04}	05
intracellular membrane-bounded organelle (GO:0043231)	1.68×10^{-03}	15
axon (GO:0030424)	2.83×10^{-03}	03
nucleus (GO:0005634)	4.23×10^{-03}	13
lysosomal membrane (GO:0005765)	1.07×10^{-02}	03
organelle inner membrane (GO:0019866)	1.22×10^{-02}	03
mitochondrial outer membrane (GO:0005741)	1.33×10^{-02}	02
asymmetric synapse (GO:0032279)	1.48×10^{-02}	02
postsynaptic density (GO:0014069)	1.58×10^{-02}	02

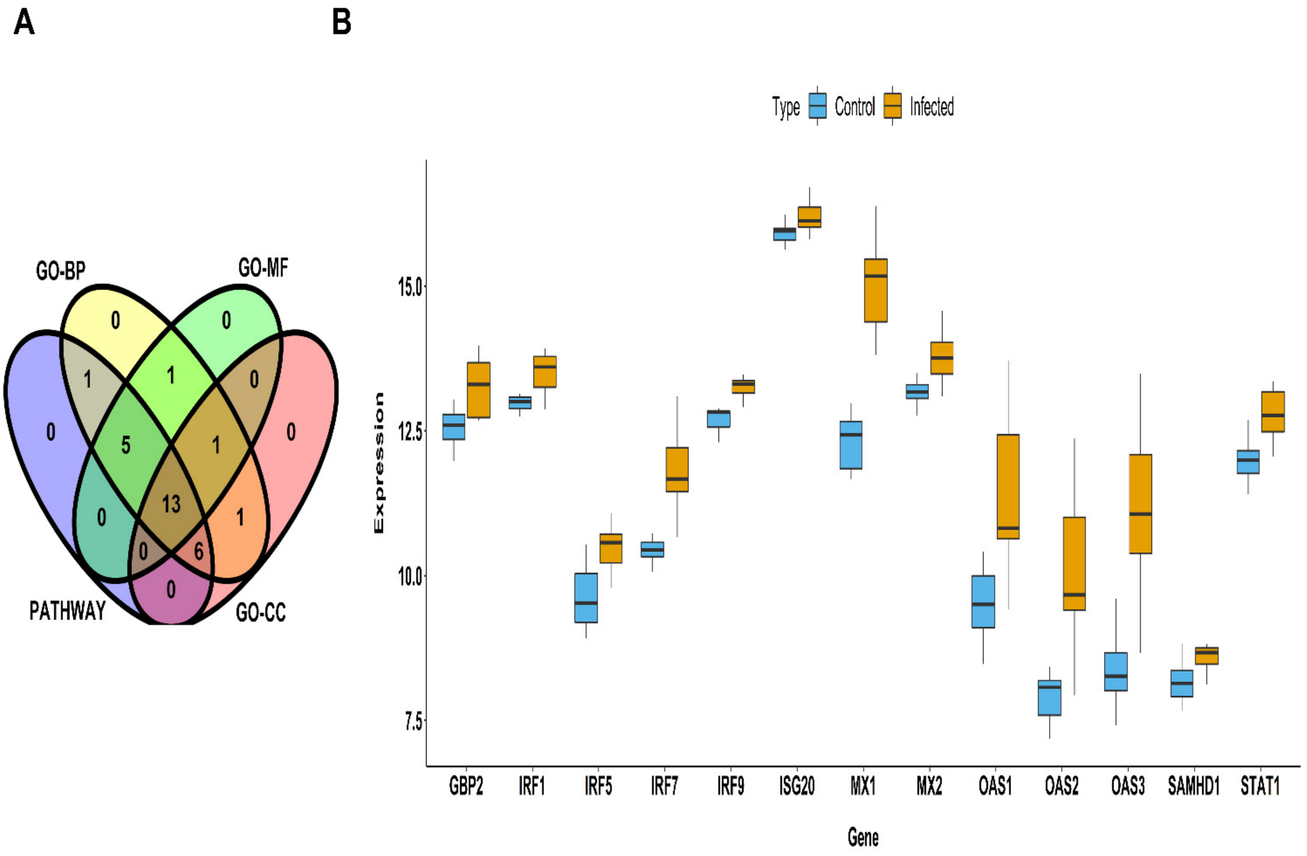

Figure S5. (A) Overlapping 13 HEV-hub genes between top 10 significant GO term and pathway genesets. The red, blue, yellow, and green colored areas signify GO-CC, BioPlanet, GO-BP, GO-BP, GO-MF genesets. **(B)** Box-and-whisker plots showing the expression intensity distribution of GBP2, IRF1, IRF5, IRF7, IRF9, ISG20, MX1, MX2, OAS1, OAS2, OAS3, SAMHD1, and STAT1 across control and infected patient WB samples. Blue and golden yellow color signify control WB and infected WB samples. The top and bottom of the boxes signify 75th and 25th percentile of distribution. Horizontal lines within the boxes represent the median values while the axes endpoints are labeled by minimum and maximum values.

HEV-specific 3-node miRNA FFL analysis

Table S7. Summary of regulatory relationships between HEV-associated mRNAs, miRNAs, and TFs.

Relationship	No. of edges	No. of miRNAs	No. of TFs	No. of mRNAs
**miRNA-mRNA[a]**	14	04	-	08
**TF-mRNA[b]**	31	-	04	08
**miRNA-TF[c]**	6	04	04	-

[a]miRNA-mRNA: miRNA repression of genes; [b]TF-mRNA: TF regulation of genes; [c]miRNA-TF: miRNA repression of TFs.

Table S8. Top 3 miRNAs ranked based on centrality measures such as degree, betweenness, closeness, radiality, and eigenvector.

miRNA	Degree	Betweenness	Closeness	Radiality	Eigenvector
**miR-222-3p**	7	4.08	0.65	0.94	0.27
**miR-214-3p**	6	6.39	0.62	0.94	0.21
**miR-125b-5p**	4	1.06	0.55	0.92	0.16

Table S9. Top 3 mRNAs ranked based on centrality measures such as degree, betweenness, closeness, radiality, and eigenvector.

Gene	Degree	Betweenness	Closeness	Radiality	Eigenvector
**OAS3**	7	9.05	0.65	0.94	0.27
**IRF5**	6	6.58	0.62	0.94	0.23
**IRF1**	6	5.85	0.62	0.94	0.24

Table S10. Top 3 TFs ranked based on centrality measures such as degree, betweenness, closeness, radiality, and eigenvector.

TF	Degree	Betweenness	Closeness	Radiality	Eigenvector
**ETV7**	10	26.49	0.75	0.96	0.33
**TRAFD1**	10	26.65	0.75	0.96	0.34
**SP140**	9	21.49	0.71	0.96	0.29

Protein-Protein docking analysis and interaction studies

Table S11. Active residues of domain 3 of OAS3 (AF_Q9Y6K5-F1) and FLN29 or TRAFD1 (PDB ID: 2D9K) proteins identified using literature and predicted using CASTp web server respectively.

Protein	Active Residues
OAS3	Asp816, Asp818, and Asp888
TRAFD1	Cys49, Gly50, Arg51, Glu68, Gly69, Ser70, Gly71, Pro72, Ser73, Ser74, Gly75

Table S12. Statistical analysis for HADDOCK generated OAS3-TRAFD1 docked complexes.

S.no.	Cluster	HADDOCK Score (in Kcal/mol)[a]	Cluster size	RMSD from overall lowest-energy structures (Å)	Van der Waals energy	Electrostatic Energy[b]	Desolvation energy	Restraints violation energy	Buried surface area (Å^2)	Z-score
1	3	-91.7 +/- 6.1	22	0.7 +/- 0.5	-53.9 +/- 4.6	-275.6 +/- 24.9	16.4 +/- 1.6	10.4 +/- 9.5	1706.5 +/- 71.8	-2.4
2	5	-69.9 +/- 0.5	10	10.6 +/- 0.4	-42.2 +/- 4.6	-194.1 +/- 29.2	3.7 +/- 1.9	74.7 +/- 9.8	1436.9 +/- 41.0	-0.6
3	1	-63.2 +/- 2.9	46	12.5 +/- 0.3	-37.5 +/- 1.3	-198.0 +/- 4.8	10.3 +/- 0.6	36.3 +/- 23.4	1381.0 +/- 28.4	-0.1
4	9	-61.4 +/- 0.5	4	10.0 +/- 0.5	-34.4	-195.4 +/- 11.6	10.2 +/- 1.5	18.0 +/- 15.2	1271.1	0.1

					+/- 4.4				+/- 59.4	
5	6	-60.8 +/- 7.6	8	12.3 +/- 0.2	-39.2 +/- 6.0	-166.7 +/- 33.0	7.9 +/- 2.6	38.9 +/- 15.3	1267.2 +/- 103.4	0.1
6	7	-60.7 +/- 6.4	6	9.9 +/- 0.3	-27.8 +/- 3.3	-244.4 +/- 27.8	12.3 +/- 2.7	37.0 +/- 22.4	1427.2 +/- 131.0	0.1
7	4	-56.6 +/- 2.3	16	8.3 +/- 0.1	-30.1 +/- 2.9	-185.2 +/- 15.2	8.1 +/- 3.3	24.4 +/- 19.5	1248.9 +/- 112.5	0.5
8	2	-52.5 +/- 2.4	27	10.7 +/- 1.8	-33.1 +/- 3.5	-150.7 +/- 31.3	6.7 +/- 3.2	40.6 +/- 23.9	1107.8 +/- 80.1	0.8
9	8	-44.8 +/- 9.5	5	5.3 +/- 1.5	-25.6 +/- 4.7	-128.7 +/- 77.0	5.4 +/- 6.4	11.8 +/- 15.9	1013.0 +/- 190.9	1.4

[a] The HADDOCK score = 1.0E_{vdw} + 0.2E_{elec} + 1.0E_{desol} + 0.1E_{AIR}

[b] Non-bonded interactions were calculated with the Optimized Potentials for Liquid Simulations (OPLS) force field using an 8.5Å cut-off.

Table S13. Interfacial residues in docked complex of OAS3-TRAFD1. Protein-protein main chain/side chain-side chain hydrogen bonds. Chain A represent OAS3_D3 protein and chain B represent TRAFD1 protein.

a. Side Chain-Main Chain Hydrogen Bonds

S.no.	Chain A	Distance (Å)	Chain B
1	^{NH1}ARG814	2.91	^{OB}CYS49
2	^{NH2}ARG814	3.05	^{OB}CYS49
3	^{NH2}ARG814	3.09	^{OB}GLY50
4	^{NE}ARG814	3.17	^{OB}GLY71
5	^{NH2}ARG814	3.10	^{OB}SER74
6	^{ND2}ASN867	3.46	^{OB}LEU58
7	^{ND2}ASN867	2.83	^{OB}LYS59
8	^{NE}ARG869	3.20	^{OB}LYS59
9	^{NH2}ARG869	3.40	^{OB}LYS59
10	^{OG}SER874	3.38	^{OB}ASN48
11	^{OD2}ASP888	2.85	^{OB}PRO62
12	^{NE2}GLN954	3.20	^{OB}ARG67
13	^{OE1}GLN954	2.97	^{OB}GLY69
14	^{OD2}ASP886	3.07	^{NB}GLY66

b. Side Chain-Side Chain Hydrogen Bonds

S.no.	Chain A	Distance (Å)	Chain B
1	^{OG}SER874	2.81	^{ND2}ASN48
2	^{OD2}ASP886	3.98	^{SG}CYS49
3	^{OD2}ASP886	2.89	^{SG}CYS65
4	^{OD2}ASP816	2.90	^{NH1}ARG67
5	^{OD1}ASP818	2.70	^{NH1}ARG67
6	^{OD1}ASP818	2.71	^{NH2}ARG67
7	^{OD2}ASP888	2.64	^{NH2}ARG67
